# Characterisation of hepatobiliary lesions in an African referral hospital: initial MDCT dose challenges

**DOI:** 10.1186/1470-7330-15-S1-P7

**Published:** 2015-10-02

**Authors:** TM Mutala, NM Tole, NM Kimani

**Affiliations:** 1 University of Nairobi, Nairobi, Kenya

## Aim

To assess the justification of abdominal CT examinations carried out, quantify radiation dose and evaluate the optimisation of scanning parameters that contribute to patient radiation dose.

## Methods

A crossectional survey was carried out on 69 patients who underwent triple phase CT imaging for suspected hepatobiliary neoplastic involvement at a referral hospital in Kenya that had acquired its first multidetector CT scan (16 slice) between July 2008 and March 2009. Justification of examination (matching clinical indications and imaging findings), optimisation of scanning parameters such as mAs and KVp as well as radiation doses in dose length product (DLP) and effective dose were assessed.

## Results

Seventy three per cent of the patients had diagnosis that was supportive of their management while 20 % had negative findings and 7% had indeterminate findings that required further imaging evaluation. The mAs and KVp were 300 and 120 respectively. DLP range was from 2633 to 3990 mGy.cm with a mean 3087.5 and standard deviation 322.81. The estimated effective dose mean was 52.38 mSv and a standard deviation of 5.49.

## Conclusion

The findings of this study acknowledge that triple phase imaging has a clear diagnostic advantage where the indication is qualified. However, more technical support is required for optimisation of imaging protocols to contain radiation doses to internationally expected levels for any new technology especially in regions where medical physicists are few.

